# THE ROLE OF FAMILIES IN FACILITATING USE OF HOME-BASED SERVICES

**DOI:** 10.1093/geroni/igac059.1644

**Published:** 2022-12-20

**Authors:** Jennifer Reckrey, Duzhi Zhao, Christine Ritche, Katherine Ornstein

**Affiliations:** Icahn School of Medicine, The Mount Sinai Hospital, New York, New York, United States; Icahn School of Medicine, The Mount Sinai Hospital, New York, New York, United States; Massachusetts General Hospital, Boston, Massachusetts, United States; Johns Hopkins University, Baltimore, Maryland, United States

## Abstract

Homebound older adults rely on home-based clinical services (e.g., home based medical care) and long-term services and supports (e.g., caregiving support, environmental modifications) to remain living at home, however patterns of service use are unknown. Using data from the 2015 National Health and Aging Trends Study linked to Medicare Claims Data, we identified a population of homebound older adults enrolled in fee-for-service Medicare (n=1066). Latent class analysis identified 3 distinct patterns of home-based service use: low (36%), medium (51%), and high (13%) service use. High family caregiving support was particularly prevalent in the medium service group (35% with over 40 hours of family care per week), but receipt of home-based medical care was minimal in this group. Additional support to connect family caregivers with other home-based services may improve care and outcomes for older adults living at home.

